# Ecological genomics in *Xanthomonas*: the nature of genetic adaptation with homologous recombination and host shifts

**DOI:** 10.1186/s12864-015-1369-8

**Published:** 2015-03-15

**Authors:** Chao-Li Huang, Pei-Hua Pu, Hao-Jen Huang, Huang-Mo Sung, Hung-Jiun Liaw, Yi-Min Chen, Chien-Ming Chen, Ming-Ban Huang, Naoki Osada, Takashi Gojobori, Tun-Wen Pai, Yu-Tin Chen, Chi-Chuan Hwang, Tzen-Yuh Chiang

**Affiliations:** Department of Life Sciences, National Cheng Kung University, Tainan, 701 Taiwan; Institute of Biotechnology, National Cheng Kung University, Tainan, 701 Taiwan; Department of Computer Science and Engineering, National Taiwan Ocean University, Keelung, 202 Taiwan; National Institute of Genetics, Mishima, Shizuoka, 411-8540 Yata Japan; Computational Bioscience Research Center, Biological and Environmental Sciences and Engineering Division, King Abdullah University of Science and Technology, Thuwal, 23955-6900 Kingdom of Saudi Arabia; Department of Engineering Science and Supercomputing Research Center, National Cheng Kung University, Tainan, 701 Taiwan

**Keywords:** Xanthomonas, Adaptive diversification, Host shift, Parapatric speciation, Homologous recombination, Comparative genomics

## Abstract

**Background:**

Comparative genomics provides insights into the diversification of bacterial species. Bacterial speciation usually takes place with lasting homologous recombination, which not only acts as a cohering force between diverging lineages but brings advantageous alleles favored by natural selection, and results in ecologically distinct species, e.g., frequent host shift in *Xanthomonas* pathogenic to various plants.

**Results:**

Using whole-genome sequences, we examined the genetic divergence in *Xanthomonas campestris* that infected Brassicaceae, and *X. citri*, pathogenic to a wider host range. Genetic differentiation between two incipient races of *X. citri* pv. *mangiferaeindicae* was attributable to a DNA fragment introduced by phages. In contrast to most portions of the genome that had nearly equivalent levels of genetic divergence between subspecies as a result of the accumulation of point mutations, 10% of the core genome involving with homologous recombination contributed to the diversification in *Xanthomonas*, as revealed by the correlation between homologous recombination and genomic divergence. Interestingly, 179 genes were under positive selection; 98 (54.7%) of these genes were involved in homologous recombination, indicating that foreign genetic fragments may have caused the adaptive diversification, especially in lineages with nutritional transitions. Homologous recombination may have provided genetic materials for the natural selection, and host shifts likely triggered ecological adaptation in *Xanthomonas*. To a certain extent, we observed positive selection nevertheless contributed to ecological divergence beyond host shifting.

**Conclusion:**

Altogether, mediated with lasting gene flow, species formation in *Xanthomonas* was likely governed by natural selection that played a key role in helping the deviating populations to explore novel niches (hosts) or respond to environmental cues, subsequently triggering species diversification.

**Electronic supplementary material:**

The online version of this article (doi:10.1186/s12864-015-1369-8) contains supplementary material, which is available to authorized users.

## Background

Diversification of prokaryotes attracts much attention from ecologists because it plays a critical role in ecosystem equilibrium and dynamics. Speciation of bacteria is distinct from eukaryotes, especially given the predominantly asexual reproduction [1] and the rare occurrence of geographic barriers. Bacterial speciation is often triggered by adaptive divergence [2,3], while homologous recombination, which leads to gene flow cohering diverging populations, simultaneously occurs, a model approximating parapatric or sympatric speciation [4,5]. Accordingly, diversifying selection plays a key role in differentiating sister species. On the other hand, favorable genes could be brought by homologous recombination enable the recipient to explore a new niche [6,7], an example of adaptive processes in bacterial diversification under gene flow [8-11].

Many of the bacteria in the genus *Xanthomonas*, of the γ-subdivision of Proteobacteria, cause plant diseases, e.g., bacterial spots and blights in leaves and fruits [12]. These plant pathogens often display a high degree of host specificity [13], e.g., *X. citri* pv. *citri* exclusively infecting citrus, with various genetic mechanisms associated with the host specificity [14]. Host shifts occurred in pv. *mangiferaeindicae* that causes bacterial black spot in mango, and in pv. *vesicatoria* that attacks pepper and tomato, displaying a wide phylogenetic range of hosts in *X. citri* [15,16]. In contrast, *X. campestris* pathovars predominantly infect crucifers [17]. It has been known that favorable traits enable deviating populations to explore novel niches in an ecosystem [18]. During habitat shifts such as host transformation, evolutionary footprints of adaptation were often reserved in the genomes [19]. Lu et al. found that six pathogenicity-related gene clusters were associated with the genomic divergences in *Xanthomonas* [20]. In this study, via a comparative genomic analysis, we comprehensively investigated genomic divergence and adaptation in *Xanthomonas,* and the contributions of host shifts and homologous recombination to the adaptive diversification between species.

To date, whole genomes of 8 *Xanthomonas* taxa, including *X. campestris* pv. *campestris* (3 strains) [21-23], *X. campestris* pv. *raphani* [17], *X. citri* pv. *vesicatoria* [24], *X. citri* pv. *citrumelo* [25], *X. citri* pv. *citri*, *X. albilineans* [26], *X. oryzae* pv. *oryzae* (3 strains) [27-29], and *X. oryzicola* [30], have been sequenced with conventional shotgun sequencing. The size of their genomes varies from 4.9 to 5.4 Mb, with a high GC content of 63.3–69.7% in the chromosome [12]. Phylogenomic analysis revealed a close affinity among *X. citri* pv. *mangiferaeindicae*, *X. citri* pv. *citri*, and *X. citri* pv. *citrumelo*, three taxa that are closely related to *X. citri.* pv. *vesicatoria* [25,31]. Recently, a 5.1-Mb genome of *X. citri* pv. *mangiferaeindicae* strain LMG 941 from India with 195 contigs was obtained by pyrosequencing [31]. In order to comprehend the genetic divergence among recently diverging strains, we sequenced the genome of BCRC 13182, a local *mangiferaeindicae* strain from Taiwan. As the two strains LMG 941 and BCRC 13182 of *X. citri* pv. *mangiferaeindicae* diverged only recently, how they became genetically differentiated is quite fascinating. In this study, for exploring the nature of adaptive diversification in *Xanthomonas*, comparative genomic analyses were conducted on two nonsister species complexes of *Xanthomonas*, i.e., *X. citri* and *X. campestris*. The patterns of genomic divergence, homologous recombination, and genes under positive selection were examined to elucidate the ecological interactions among *Xanthomonas* taxa.

## Results

### General features of the *Xanthomonas citri* pv. *mangiferaeindicae* genome

**Figure 1:**
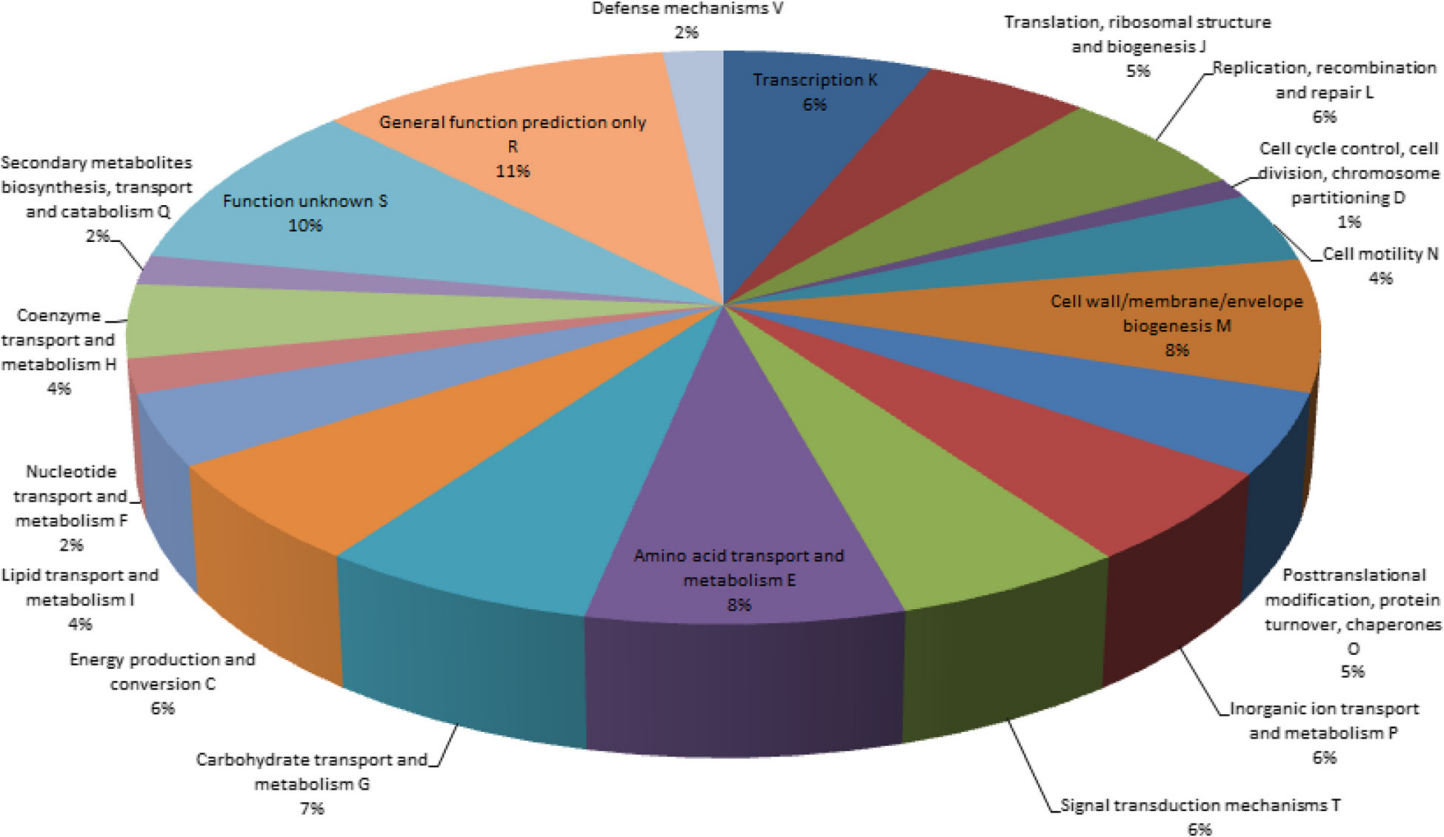
**Functional categories of annotated genes in the genome of** ***Xanthomonas citri*** **pv.** ***mangiferaeindicae*** **BCRC 13182.** Colors on the pie chart represent predicted clusters of orthologous groups (COG).

The genome of *X. citri* pv. *mangiferaeindicae* BCRC 13182 (XCM-B) was sequenced, and 4,292,719,080 bases of paired-end data (read length = 60 bp, Q30 percentage = 98.8%) and 1,185,355,594 bases of mate-paired data (read length = 101 bp, Q30 percentage = 77.0%) were retrieved. These sequences were *de novo* assembled to 221 contigs, and scaffolded into 43 scaffolds, which comprised 5,355,324 bp with a GC content of 64.75% and indicated the sequence coverage is 1022.92. The largest scaffold was 1,286,619 bp long, and the N50 statistic was 549,958 bp, with an average length of 124,542 bp (Table 1). The protein-coding gene prediction, confirmed by BLAST searches against the NCBI database, identified 5,362 coding sequences, 3,837 of which could be categorized into clusters of orthologous groups (COG) (Figure 1). When compared with the draft genome of XCM strain LMG 941 (XCM-L) (Table 1), these two strains shared similar GC contents and 9 rRNA genes in 3 sets of rRNA operon (16S–23S–5S), whereas the average length (124,542 vs. 26,213), N50 statistic (549,958 vs.67,371), number of protein-coding sequences (5,362 vs. 4,521), and number of tRNA genes (55 vs. 51) were all greater in XCM-B, suggesting higher genome completeness in the genome that we sequenced and assembled. In addition, we aligned 43 scaffolds of XCM-B against those of XCM-L. Among the 195 contigs of XCM-L, 126 could be mapped onto the 18 scaffolds of XCM-B, whereas none of the XCM-L contigs were mapped onto XCM-B with more than one scaffold (Additional file 1: Table S1). Nevertheless, aligning both sequences helped verify the conserved genomes between the two strains. The genomes of two XCM strains shared 4,074 protein-coding genes, with only 203 variables, reaching an average protein sequence identity of 99.89%.

**Table 1:** **Genome features of** ***X. citri*** **pv.** ***mangiferaeindicae*** **strains BCRC 13182 (XCM-B) and LMG 941 (XCM-L)**

**Chromosome feature**	**XCM-B**	**XCM- L**
**General feature of the genomes**		
Scaffold numbers	43	195
N50 size (bp)	549,958	67,371
Average length (bp)	124,542	26,213
Genome size (bp)	5,355,324	5,111,537
G + C content (%)	64.75	64.85
**Predicted CDSs**		
Protein-coding genes	5,362	4,521
With assigned to COGs	3,837	NA
**Shared protein-coding genes**	4074	
Identical amino acid sequences	3871	
Sequences with indels	83	
**Noncoding genes**		
rRNA	9	9
rRNA operons	3	3
tRNA	55	51

NA, not available.

### Phylogenetic relationships and homologous recombination among *Xanthomonas* species

**Figure 2:**
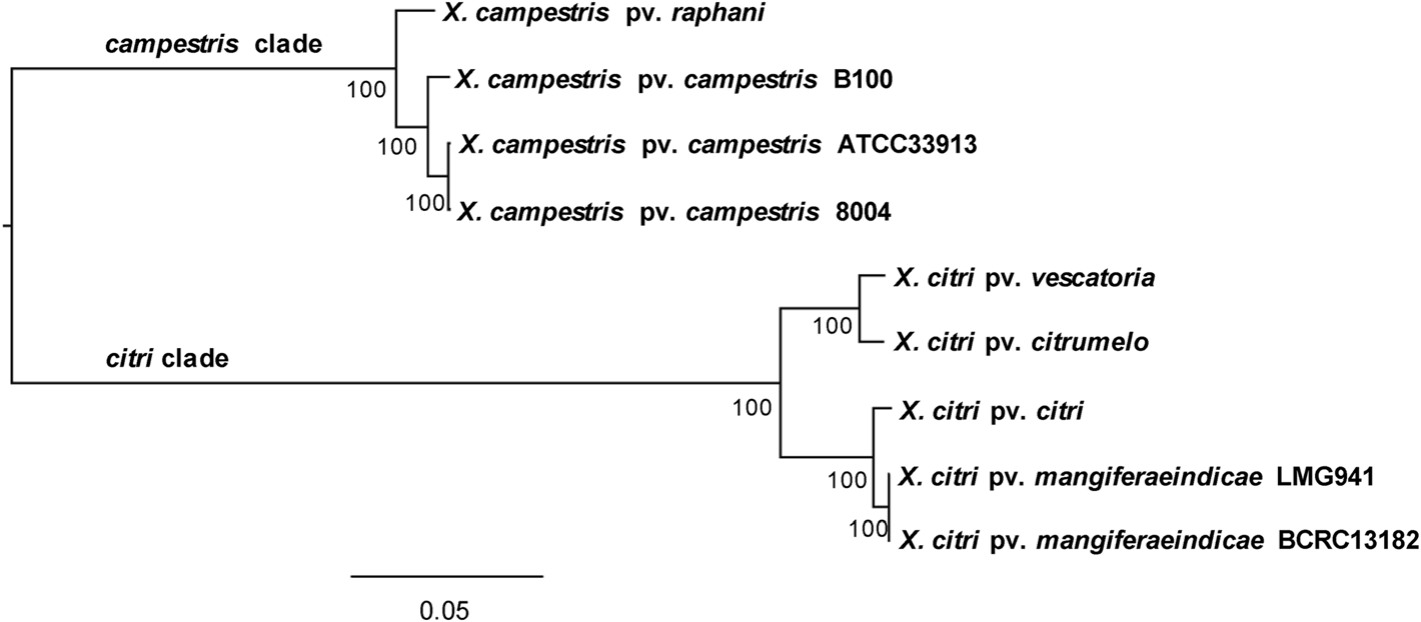
**Molecular phylogeny of 9** ***Xanthomonas*** **taxa based on 2851 core orthologous genes.** Numbers at the nodes represent bootstrap values.

As XCM-B that infects mango was once classified as a member of *X. campestris* [32], we tested the robustness of the classification of 5 *X. citri* taxa (XCM-B, XCM-L, *X. citri* pv. *citri* [XCC], *X. citri* pv. *citrumelo* [XCCM], *X. citri* pv. *vesicatoria* [XCV]) and 4 *X. campestris* taxa (*X. campestris* pv. *campestris* ATCC33913, 8004, B100 [XCCA, XCC8, XCCB], *X. campestris* pv. *raphani* [XCR]) (Additional file 2: Table S2). Based on a concatenated alignment of 2851 orthologous core genes shared by the 9 taxa, the best maximum likelihood (ML) tree identified two major clades corresponding to species. In the *citri*-clade, 2 XCM strains were clustered with XCC, while XCCM was sister to XCV; within the *campestris*-clade, XCCA and XCC8 were closely related, both being affined to XCCB and XCR (Figure 2).

Recombination can cohere the taxa within a species, but dissociates sister taxa when it occurs between long-split species. Here, we combined the alignment-based programs geneconv and PhiPack to detect genes affected by recombination between or within species. In total, 283 core genes (9.9% of the genome) were detected with genetic recombination, which included 83 genes with cross-overs occurring between *X. citri* and *X. campestris*, 172 genes only detected within species, while 28 have likely derived from other distant species (Additional file 3: Table S3). Single gene trees were generated with the neighbor-joining method. For 2786 of the 2851 trees (97.7%), the topologies agreed with the division between *citri* and *campestris*, suggesting a long split between the 2 species.

### Genome-wide variations and recombination events

**Figure 3:**
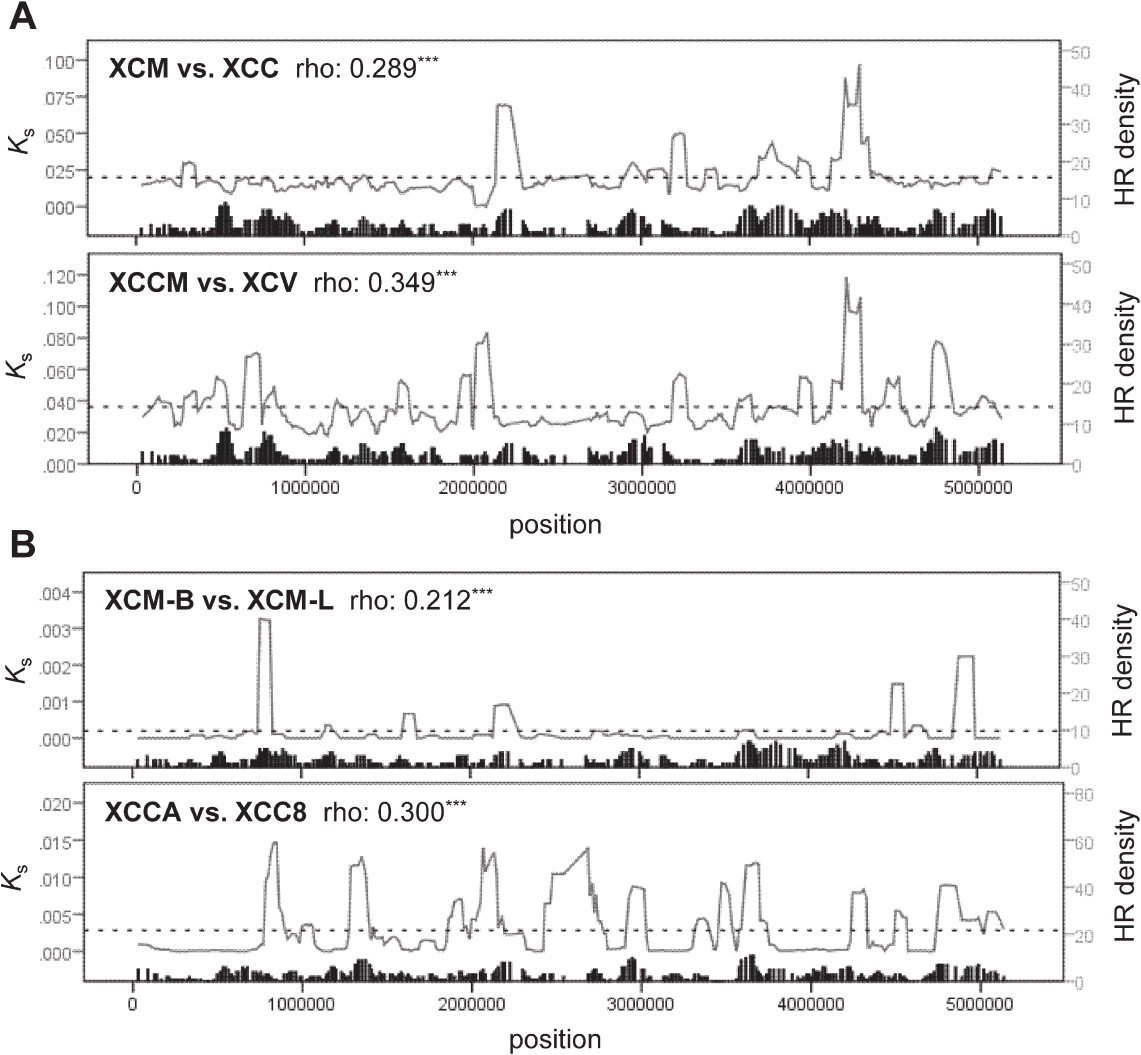
**Genome-wide distributions of** ***K***_s_ **divergence and the density of homologous recombination (HR) events.** *K*s mean values and the density of homologous recombination events were plotted with a 50-gene window along the *X. citri* pv. *citri* (XCC) genome. The asterisks on rho value denote significant Spearman’s correlation (***, P < 0.001). Dashed lines represent the mean of *K*_s_ values. **A.** Two host-shifting pairs: XCC vs. *X. citri* pv. *mangiferaeindicae* BCRC 13182 (XCM-B), between citrus and mango; *X. citri* pv. *citrumelo* (XCCM) vs. *X. citri pv. vescicatoria* (XCV), between citrus and mango. **B.** Two closely-related pairs: XCM-B vs. *X. citri* pv. *mangiferaeindicae* LMG 941 (XCM-L); *X. campestris pv. campestris* ATCC 33913 (XCCA) vs. *X. campestris pv. campestris* 8004 (XCC8).

Genetic divergences were not homogeneously distributed along the chromosome. Some genomic regions displayed higher divergences than the others, as so-called “genomic islands of divergence” [33]. Here, we used synonymous substitution rates (*K*_s_), a near-neutral indicator of genetic divergence, to assess the divergence along the genome. In the comparison between 2 XCM strains based on the orthologous genes between XCM and XCC, only one major peak was observed in the putative prophage region, while the remaining regions displayed more or less uniform levels of genomic variation (Additional file 4: Figure S1). We further investigated the genic structure of the prophage region. Of the genes shared between the two strains of XCM, 53 remained intact, while 18 genes had become eliminated (see Additional file 5: Figure S2A). In the comparison between XCM-B and XCC, 33 genes were lost in XCM-B (Additional file 5: Figure S2B). All these results supported rapid evolution with dramatic gain or loss of genes in the prophage region. Furthermore, to examine the associations between host shifting and genomic divergence, pairwise comparisons were conducted in pairs of XCM vs. XCC (shifting between citrus and mango) and XCCM vs. XCV (between citrus and pepper) using 2851 core orthologous genes. Two major *K*_s_ peaks of 3.20–3.25 M bp and 4.20–4.35 M bp were shared by the host-shifting pairs, while only a few recombination events were detected in these regions (Figure 3A). It is evident that numerous divergence peaks overlap with peaks of the density of recombination events, implying a correlation between homologous recombination and genetic divergence (Figure 3A). As shown by Spearman’s rank correlation test, both comparisons were significant in the correlation between *K*_s_ values and the number of genes with recombination (Spearman’s rho = 0.29–0.35, *P* < 0.001). In addition, comparisons between closely related strains, i.e., XCM-B vs. XCM-L and XCCA vs. XCC8, were also performed. Similar to the comparisons of host-shifting pairs, significant positive correlations were also observed in the strain pairs without host-shifting (Spearman’s rho = 0.21–0.30, both *P* < 0.001, Figure 3B). At the gene level, the genes with recombination showed higher *K*_s_ than those without recombination based on pairwise comparisons (Mann–Whitney *U* test, *P* < 0.001) (Additional file 6: Figure S3), implying that these genes may facilitate the diversification among *Xanthomonas* strains. Taken together, these results suggest that homologous recombination largely affect the pattern of genomic divergence between *Xanthomonas* species*.*

### Genes under positive selection

**Figure 4:**
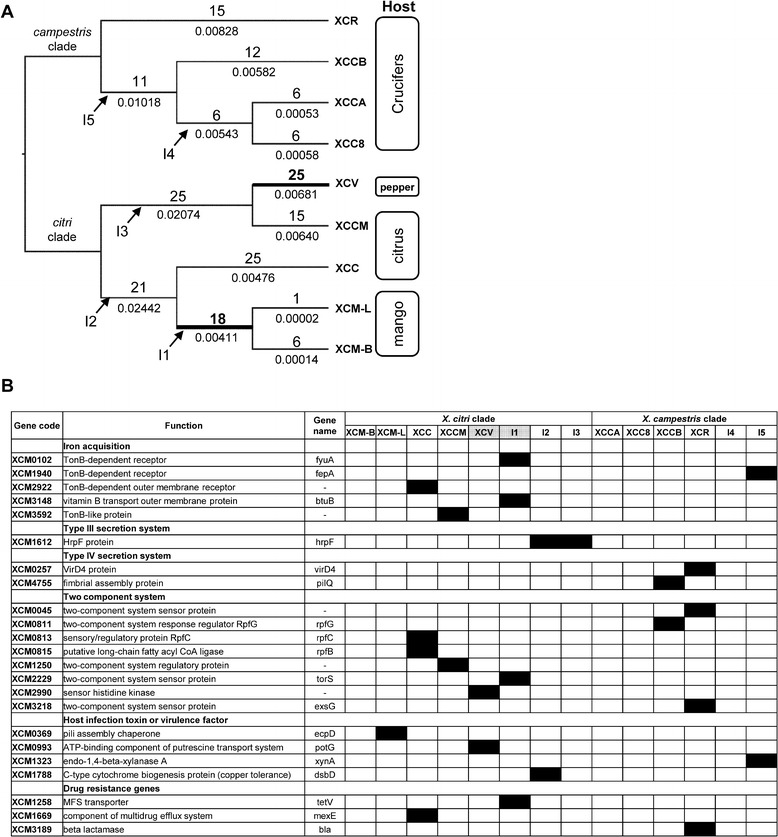
**Summary of positively selected genes. (A)** Distribution of positively selected genes in the phylogenetic cladogram. The numbers above and under the branches denote the number of positively selected genes and branch lengths, respectively. Thick lines and bold numbers denote the occurrences of host shift events. Internal branches are labeled from I1 to I5 with arrows. **(B)** Positively selected genes related to plant pathogenicity. Putative functions were based on the gene annotations of *X. citri* pv. *citri*. Genes under positive selection are filled in black at the corresponding branch, which is denoted with taxon name (terminal branch) or codes I1–I5 (internal branch). Host-shift branches were labeled in grey.

For identifying positively selected genes within each *Xanthomonas* lineage, codeml analyses were conducted with the branch-site model on all branches. In total, 179 genes (6.3% of the core genome) were detected under positive selection. Of these positively selected genes, only 3 were shared between *X. citri* and *X. campestris*, while 125 were exclusively in *X. citri*, and 51 were only in *X. campestris* (see Additional file 7: Table S4), implying different diversification scenarios.. Among the tree branches of the *citri*-group, along the branches of XCV and XCM, with host shifts, 25 and 18 positively selected genes were detected, respectively (Figure 4A). As for the remaining branches without host-shift events, the numbers of positively selected genes are as follows: 1 in XCM-L, 6 in XCM-B, 15 in XCCM, and 25 in XCC. Likewise, in the *campestris* group, 6 positively selected genes occurred in the XCCA lineage, followed by 6 in XCC8, 12 in XCCB, and 15 in XCR. Interestingly, the lineages in *citri*-group coupled with the host-shift events possessed more genes under positive selection (P < 0.001, g-test). We further tested the association between homologous recombination and positive selection. In total, 98 (57%) out of 184 positively selected genes were identified with recombination, with 73 in the *citri*-group, and 26 in the *campestris*-group, all significantly deviating from a random distribution (*P* < < 0.001, Fisher’s exact test) (Table 2). Intriguingly, the majority of the genes under positive selection did not display tree topologies deviating from the species tree, as shown by 98.4% (126/128) in the *citri*-group and 96.3% (52/54) in the *campestris*-group agreeing with the species tree (both *P* > > 0.05, Fisher’s exact test) (Table 3).

**Table 2:** **Distributions of the genes with recombination and the genes under positive selection in** ***Xanthomonas***

		**Positively selected**	**Non-positively selected**	**Fisher’s exact test**
***X. citri***				
	**With recombination**	73	210	***P*** **< 0.0001**
	**Without recombination**	55	2513	
***X. campestris***				
	**With recombination**	26	257	***P*** **< 0.0001**
	**Without recombination**	28	2540	
**Total**				
	**With recombination**	98	185	***P*** **< 0.0001**
	**Without recombination**	81	2487

**Table 3:** **Distributions of positively selected genes and tree topologies in** ***Xanthomonas***

		**Separation of species groups**		
		**Agree**	**Disagree**	**Fisher’s exact test**
***X. citri***				
	**Positively selected**	126	2	***P*** **= 1**
	**Non-positively selected**	2660	63	
***X. campestris***				
	**Positively selected**	52	2	***P*** **= 0.3503**
	**Non-positively selected**	2734	63	
**Total**				
	**Positively selected**	175	4	***P*** **= 1**
	**Non-positively selected**	2611	61	

Of the numerous genes putatively under the positive selection, 23 loci were likely to be associated with the plant pathogenicity [34-37] (Figure 4B). For example, 5 genes were involved in the iron acquisition, especially under the environment with the low availability of free iron from the host [38]. Of these genes for protein secretion systems that help the transportation of virulence factor from bacteria to host, 4 loci were exclusively favored in the *citri* lineage that with 1 genes of the type III, and 2 gene of the type IV [36,37]. Besides, 8 genes related to the two component system of bacteria, which acts as a sensor to environmental cues and an activator of pathogenic genes; 3 genes were associated with the antibiotic resistance, and 4 other genes were related to the various functions with the plant pathogenicity. For the positively selected genes that might have ecological impacts, we listed them in Table 4 and fully discussed in the next section.

**Table 4:** **Ecological effects of positively selected genes**

**Positively selected genes**	**Selected lineage**	**Related to host shift**	**Related to homologous recombination**	**Putative ecological effects**
RpfB, RpfC (XCM0813, XCM0815)	*X. citri* pv. *citri*	Yes	Yes	Altering pathogenicity to favor exploration of different host
DsbD (XCM1788)	I2 branch	No	Yes	Increasing copper tolerance in escaping from copper-containing bactericides
PotG (XCM0993)	*X. citri pv. vesicatoria*	Yes	Yes	Reducing AMF colonization of host plant and increasing infectivity
BtuB (XCM3148)	I1 branch (*X. citri* pv. *mangiferaeindicae*)	Yes	No	Increasing iron uptake with host-shifting from citrus to mango
HrpF (XCM1612)	I2, I3 branches	Yes	Yes	Controlling invasive routes and host specificity of *X. citri*
EcpD (XCM0369)	XCM-L	No	No	Helping pili assembly and associating with virulence
XynA (XCM1323)	I5 branch	No	No	Breaking down xylan and causing virulence

## Discussion

### Speciation processes in Xanthomonas

Bacterial diversity, which often results from species diversification through ecological interactions, greatly influences the ecosystem health. *Xanthomonas* species, which can cause serious diseases and economic loss of crops, are an excellent group for examining speciation models. The genomes of 8 taxa that belong to *X. citri* and *X. campestris* have been completely sequenced, providing a large data set as a sound foundation for investigating the extent of genetic divergence between species. Their host specificity makes the group even more useful as a system for examining adaptation over the host shifts because the host range of the group as a whole is quite broad. In this study, two strains of *X. citri* pv. *mangiferaeindicae* from Taiwan (XCM-B) and India (XCM-L) and two strains of *X. campestris* (XCCA and XCC8) displayed slight genetic differentiation, providing a window for investigating incipient genetic divergence. As expected, given a shorter time for isolation, strains had lower levels of genetic divergence than that of species/pathovars. It is noticeable that prophage-introduced DNA fragments were detected in *X. citri* pv. *mangiferaeindicae* and pv. *citri* [39], an event that exclusively occurred in *X. citri*. Several salient features were observed in these inserted DNA fragments. First, long DNA fragments of 28,787 bp were lost in genomes of the two pathovars (Additional file 5: Figure S2). Second, a high proportion of genes (18 of 71 between strains of *mangiferaeindicae*, and 33 of 71 between pathovars) became eliminated along with the divergence time. Third, higher nucleotide substitution rates were detected in these inserted DNAs than in the host bacterial genome. These facts indicate that the foreign genes introduced by the phage tended to lose functions and were likely to be removed from the genome eventually (e.g., gene XCM0436), although gene residues may still remain [40].

On the other hand, homologous recombination had started to contribute noticeably to the global diverging patterns among *Xanthomonas* strains at the incipient stage, as shown by the positive and significant correlation with genomic divergence (Figure 3). Homologous recombination is well documented in prokaryotes with foreign genes introgressed into the recipient species [41]. Recombination often occurs between closely related bacteria, resulting in convergence of two bacterial populations; recombination with divergent bacteria brings variations into the recipient population, facilitating the differentiation among incipient species [42,43].

Furthermore, 1 and 6 genes under positive selection were detected in the lineages leading to XCM-L and XCM-B, respectively. Also, of the 7 genes involved in diversifying selection of pv. *mangiferaeindicae*, 3 genes were involved in homologous recombination. Similarly, 6 of 9 genes under positive selection were detected with recombination for XCCA and XCC8. At early stages of speciation or diversification, the interplay between natural selection and homologous recombination clearly played a key role in differentiating incipient species/races.

### Homologous recombination in Xanthomonas

In this study, we found frequent genetic recombination in *Xanthomonas*, with about 10% of the core genome mediated with homologous recombination, whereas only 2% of the core genes displayed phylogenies deviating from the species tree. This gap to the expectations simply came from intraspecific crossovers, which constitute the most recombination events (about 88%) with short DNA fragments. Apparently, those genes displaying trees inconsistent with the species tree represented footprints of gene flow between species. This result suggests that the divergence of *Xanthomonas* tends to follow parapatric speciation, in which gene flow between species can be long lasting and nonstop [41]. This is a pattern well documented in the Archaea and Bacteria [1,7]. It is intriguing that most of the foreign genes were favored by natural selection (Table 2), especially those associated with plant infection (Figure 4B), reflecting a fact that homologous recombination may create advantageous new alleles for a so-called “hopeful monster” [44] in bacteria. Altogether, the topology of the putative species tree in *Xanthomonas* reflects a scenario of deep divergence between species, mediated by recurrent gene flow at the same time [16].

In this study, 98 out of 283 genes with recombination were under positive selection (Table 2). Via homologous recombination, genes were able to be interchanged between strains, and advantageous alleles that helped bacterial colonization would be favored by natural selection [3,45]. Of these genes, it is noticeable that *RpfB* (XCM0815) and *RpfC* (XCM0813) of the *Rpf* gene cluster, which regulates the virulence of *Xanthomonas* [20,46,47], were positively selected in *X. citri* pv. *citri* lineage (Table 4). Functionally, *RpfB* encodes a fatty acyl-CoA ligase, which catalyzes the synthesis of an important signal molecule that regulates the expression of virulence genes [48,49]. *RpfB* signaling is perceived by the two component system of *RpfC* and *RpfG* [50], and the mutations of *RpfC* were found to reduce the pathogenicity of *X. citri* pv. *citri* [14]. Accordingly, existence of favorable *RpfB* alleles suggests that homologous recombination may have helped the adaptation of *Xanthomonas*. Besides, *DsbD* (*XCM1788*) has been reported to be associated with copper tolerance [51]. Copper compounds are frequently used as bactericides in controlling the leaf infection of *Xanthomonas* species, including *X. citri* [52,53]. A previous experiment showed that a *DsbD* knockout mutant was highly sensitive to environmental copper [54]. An effective *DsbD* allele was capable of reducing the impacts of the copper-containing bactericides, thus enabling the bacterium to escape from the agricultural control.

### Host shifts likely triggering genetic adaptation

Like the findings of previous studies, compared with the vast portions of the genome that are usually shaped by purifying selection, adaptive genes only constitute a relatively smaller proportion of the chromosome instead [55]. Furthermore, in this study, genes under positive selection (only 6.3% of the core genome) were unequally distributed along lineages, with significantly more genes located at lineages leading to nodes coupled with host shifts (Figure 4A). For example, at the lineages from *X. citri* pv. *citri* leading to *X. citri* pv. *mangiferaeindicae* and *X. citri* pv. *vescatoria* with hosts shifts among citrus, mango and pepper, respectively, 18 and 25 positively selected genes were detected, revealing faster accumulation of positively selected genes than other lineages without host shifts. However, the lineages of *X. campestris* pv. *raphani* and pv. *campestris*, both infecting the Brassicaceae without host shifting, did not show facilitated positive selection. This contrasting pattern suggested that favorable genes may have helped the bacteria in exploring new niches. The tight association between positively selected genes and host shifts further suggests that *Xanthomonas* likely followed ecological speciation, which describes species arising from ecological diversification [3].

We found that some positively selected genes were associated with the host shifting events. It is notable that the *PotG* gene (XCM0993), that encodes ATPase involved in putrescine transport, was shaped by positive selection in the *X. citri* pv. *vesicatoria* lineage. Putrescine is a ubiquitous polyamine that enhances the interaction between plants and arbuscular mycorrhizal fungi (AMF) [56], which in turn have been shown to activate plant defense against infectious pathogens such as *Xanthomonas* [57]. Alternatively, the removal of exogenous putrescine from the soil may impede host colonization by AMF; thus, an effective *PotG* gene that decreases AMF colonization of host plant will be favored by natural selection, subsequently helping *X. citri* pv. *vesicatoria* to explore a new niche (host). In addition, *BtuB* gene (*XCM3148*) was positively selected in *X. citri* pv. *mangiferaeindicae* lineage. *BtuB* is responsible for the iron uptake, while iron dependent superoxide dismutases are vital in inhibiting the reactive oxygen species (ROS) responses in host cells, thus increasing the infection rates [46]. Interestingly, previous studies revealed that the iron contents in mango leaves might be hundreds times lower than that in citrus leaves (0.27 vs. 41.7 mg kg^-1^ dry weight) [58,59]. The sharp difference suggested that an effective iron transporter was needed when the host shifted from citrus to mango.

In addition to the genes related to the host shifting, an *Hrp* regulon gene *XCM1612* that encodes components for the type III secretion system (T3SS) [60] was detected as loci under positive selection in the X. *citri*. The pathogenicity of *Xanthomonas* mainly depends on the T3SS, which is highly conserved among plant and animal pathogenic bacteria [61,62]. Curiously, the amino acid sequences of the major subunit of *Hrp* pili are hypervariable in different subspecies of bacterial pathogens [63,64]. The rapidly evolving *Hrp* pili provide evidence for strong positive selection in *Xanthomonas* spp. and diversifying selection in *Pseudomonas syringae* [65,66]. The acquisition of the *Hrp* gene cluster has been found to be associated with the adaptation and plant pathogenicity in *Xanthomonas* [20]. Thus, we hypothesized that the positive selection on *HrpF* gene may be responsible for the invasion routes and the host specificity of *Xanthomona*s. Furthermore, two genes involved in type IV secretion system were detected with positive selections exclusively in *X. campestris*, seemingly agreeing that type IV secretion system may not be involved in the infection of *X. citri* on citrus [67]. Moreover, *EcpD* (XCM0369) that encodes an adhesion protein to help pili assembly was positively selected in XCM-L. *EcpD* has been shown to facilitate the polymerization of *EcpA* to form pilus in *Escherichia coli* and involve in host cell recognition or biofilm formation [68]. *Xanthomonas* species only possessed *EcpD* while lacks other members of *Ecp* operon. We therefore hypothesized that *EcpD* might participate the assembly of the other pili in association with the virulence in *Xanthomonas*. On the other hand, a xylanase gene *XynA* (XCM1323) was positively selected at the branch leading to *X. campestris* pv. *campestris* (Figure 4B, Table 4*).* Xylan is a major component in the cell walls of land plants and exists in all plant tissues [69]. Previous studies showed xylanases are responsible for the virulence of *X. citri* pv. *vesicatoria* and *X. oryzae* pv. *oryzae* [70,71]. Two gene clusters *xcs* and *xps* of the Type II secretion system (T2SS) have been shown to control the secretion of xylanases in XCV and are associated with the virulence [70]. Nevertheless, no member of these two gene clusters was detected under positive selection in this study (Additional file 3: Table S3).

## Conclusions

In this study, we sequenced a genome of *X. citri* pv. *mangiferaeindicae* and conducted genomic analyses of 9 taxa of *X. citri* and *campestris*. Between the 2 strains of XCM, only the prophage region displayed sharp differentiation, while gradually losing the constituting genes. In addition, we found homologous recombination frequently occurring in the *Xanthomonas* genomes, which likely represented footprints of gene flow between species, thus most likely suggesting parapatric speciation. It is noticeable that facilitated accumulations of positively selected genes occurred along the lineages with host shifts. Interestingly, most of the favored genes were acquired from homologous recombination. Taken together, the genes with recombination enabled *Xanthomonas* species to explore novel niches and respond to environmental stresses, subsequently resulting in adaptive diversification in this pathogenic genus.

## Methods

### Whole sequencing, assembly, and annotation of *Xanthomonas citri* pv. *mangiferaeindicae*

Bacterial strain BCRC 13182 (NCHUPP Xma1) from Taiwan of *X. citri* pv. *mangiferaeindicae* was purchased from the Food Industry Research and Development Institute and sequenced in this study. The whole genome was sequenced using a high-throughput sequencing technique with Illumina GA IIx and HiSeq sequencers [72]. Paired-end sequence reads of 60-bp were obtained, with an average distance of 150 bp between pair reads. Mate-paired sequence reads of 100 bp were obtained with an average distance of 3000 bp between pair reads. All raw sequences were stored in NCBI SRA database (SRP049288). A total of 110,671,234 and 15,543,685 high-quality sequences of 60 and 100 bp, respectively, were assembled into contigs using the *de novo* assembler ABySS version 1.2.7 [73]. After contigs shorter than 200 bp were removed, 221 contigs were generated. Scaffolding was performed with these contigs using SSPACE v2.0 [74], and 43 scaffolds were built based on the distance information between paired reads.

The draft genome was analyzed using an integrated annotation pipeline glued by the perl programming language. Glimmer version 3.02 [75] was used to predict the protein-coding regions, which were annotated with BLAST [76] against the nonredundant protein database (http://ncbi.nlm.nih.gov) and the Clusters of Orthologous Groups (COG) database [77] with a cutoff of E-value < 1 × 10^-5^. All protein-coding regions were manually curated with the EMBOSS analysis package according to the BLAST results [77]. We used tRNAscan-SE and RNAmmer version 1.2 to predict the prokaryotic transfer RNA genes and the ribosomal RNA genes [78,79].

### Comparative genomics analysis and orthologous protein identification

For comparative analyses, we downloaded 8 bacteria genomes from the NCBI GenBank (http://www.ncbi.nlm.nih.gov/genbank/) in addition to the newly assembled genomes of *X. citri* pv. *mangiferaeindicae* BCRC 13182, including *X. citri* pv. *citri* 306, *X. citri* pv. *citrumelo* F1, *X. citri pv. vesicatoria* 85–10, *X. campestris* pv. *campestris* ATCC 33913, *X. campestris* pv. *campestris* 8004, *X. campestris* pv. *campestris* B100, *X. campestris* pv. *raphani* 756C, and a draft genome of *X. citri* pv. *mangiferaeindicae* LMG 941 (Additional file 1: Table S1). Orthologous genes in these genomes were identified using bidirectional best hits (BBH) in a BLAST search [80] based on the criteria set at E-value < 1 × 10^-5^, identity > 60%, and a threshold of 70% of orthologous alignment length. The core genome with 2,851 orthologous genes and common proteins shared by *Xanthomonas* species were recognized. In total, 43 scaffolds of the *X. citri* pv. *mangiferaeindicae* strain BCRC 13182 were aligned against the strain LMG 941, which has 195 contigs. We used BLAST for pairwise nucleotide alignment of the two draft genomes.

### Phylogeny of *Xanthomonas*

For aligning nucleotide sequences of protein-encoding genes without interrupting codons, the protein sequences were first aligned by ClustalW2 [81], and the corresponding nucleotide sequences were aligned accordingly using PAL2NAL [82]. A maximum-likelihood (ML) phylogeny based on the concatenated 2851 genes was generated with the program RAxML [83]. We used the GTRGAMMA substitution model suggested by jModelTest 2.1.4 [84,85]. Confidence in the internal nodes of the ML tree was tested with 1000 rapid bootstrap replicates. For each gene phylogeny, we used ClustalW2 to generate a neighbor-joining tree with default settings.

### Detection of genetic recombination

Putative genetic recombination events occurring in *Xanthomonas* genomes were examined by using the software geneconv [86] and PhiPack [87]. First, the alignments of the 2851 orthologous genes were tested with geneconv, and a recombination event can be recognized when a Bonferroni corrected KA p-value is less than 0.05. Notably, the geneconv program is able to distinguish a recombination occurring within or between species groups. In addition, to reduce the probability of detecting false positive when merely using a single method, we used the PhiPack program, which performs three different methods (Neighbor similarity score, MaxChi, and Phi) for identifying genetic recombination, to confirm the results of geneconv. Using the PhiPack program, recombination was identified for p-values lower than 0.05. Taken together, a gene with recombination was recognized when recombination was detected with geneconv and at least two additional methods implemented in PhiPack.

### Divergence estimation

Numbers of substitutions per synonymous site (*K*_s_) and per nonsynonymous site (*K*_a_) were calculated using codeml program implemented in the PAML package with the codon-based model [88]. All core orthologous genes were mapped onto the XCC genome to perform window-sliding analyses. The genome-wide comparisons of *K*_s_ values and the number of genes with recombination were made with a window size of 50 genes and an overlap of 10 genes. Spearman’s rho coefficient was used to examine the correlations between Ks values and the number of the genes with recombination. In addition, the significant of difference of *K*s distributions between genes with and without recombination were evaluated with Mann–Whitney *U* test. Both tests were performed by using SPSS Statistics 17.0.

### Detection of genes under positive selection

For detecting genes under positive selection, we applied the codeml program from the PAML package to all core genes. The multiple alignments were fit to the F3X4 model of codon frequencies, and the branch-site alternative model (model = 2, NSsites = 2) was adopted [89]. Three independent simulations were performed with initial omega (*K*_a_/*K*_s_) values of 0.5, 1, and 1.5, respectively, while the null model was fixed with the omega value at 1. Likelihood ratio tests were used to assess the significance between the test and null models, and the *P* values were adjusted with a false discovery rate (FDR) of 0.01 for multiple testing corrections. We performed codeml analyses on each branch of individual gene trees. For above three tests, branches detected with significance were deemed to be affected by positive selection. Assuming the number of positively selected genes was proportional to the branch length, we used a chi-square test to examine the homogeneity in the distribution of positively selected genes between branches with and without host shifts. Fisher’s exact tests were also used to assess the differences of the number of positively selected genes between outlier and background genes, as well as between the genes with and without homologous recombination.

### Availability of supporting data

Alignments and phylogenetic trees of the core orthologous genes were deposited in TreeBASE **(**http://treebase.org/treebase-web/home.html**).**

## Additional files

Additional file 1: Table S1. Comparisons of assembled scaffolds and lengths between strains BCRC 13182 and LMG 941 of *X. citri* pv. *mangiferaeindicae.* (XCM). Additional file 2: Table S2. Information on 9 *Xanthomonas* species. Additional file 3: Table S3. Summary of orthologous genes among 9 *Xanthomonas* taxa. Additional file 4: Figure S1. Distribution of mean of *K*_s_ of *Xanthomonas citri* pv. *mangiferaeindicae* BCRC 13182 (XCM-B) vs. *Xanthomonas citri* pv. *mangiferaeindicae* LMG941 (XCM-L). Additional file 5: Figure S2. Schematic presentation of the putative prophage region among two *Xanthomonas citri* pv*. mangiferaeindicae* (XCM) strains and *Xanthomonas citri* pv*. citri* (XCC). Additional file 6: Figure S3. Comparisons of *K*_s_ values between the genes with and without recombination. Additional file 7: Table S4. List of positively selected genes in *Xanthomonas.*
