# Supplementary material for: Ecological genomics in Xanthomonas: the nature of genetic adaptation with homologous recombination and host shifts
Source: BMC Genomics. 2015 Mar 15;16(1):188. doi: 10.1186/s12864-015-1369-8 (PMC4372319; doi:10.1186/s12864-015-1369-8)
Supplement: Additional file 1: Table S1. — Comparisons of assembled scaffolds and lengths between strains BCRC 13182 and LMG 941 of X. citri pv. mangiferaeindicae. (XCM). [file 12864_2015_1369_MOESM1_ESM.doc]

**Table S1 Comparisons of assembled scaffolds and lengths between strains BCRC 13182 and LMG 941 of *X. citri* pv. *mangiferaeindicae*** (XCM).

| **XCM-B** | **XCM-L** | **Length (bp)** |
| --- | --- | --- |
| Scaffold 1 | Contig 18, 80, 117, 40, 18, 97, 57, 17, 8, 13, 29, 105, 20, 103,91,23, 14, 64, 76, 41, 21, 61, 102, 28, 71, 83, 122, 38 | 1,286,619 |
| Scaffold 2 | Contig 15, 77, 49, 46, 56, 50, 19, 24, 6, 121, 70,63, 4, 82, 88, 16, 33, 47, 26, 108, 9 | 1,152,948 |
| Scaffold 3 | Contig 68,12, 54, 72, 60, 52, 10, 119, 111, 79, 109, 87, 95, 99, 7 | 549,958 |
| Scaffold 4 | Contig 93, 74, 96, 126, 130, 45, 48, 59, 3, 81, 58, 120, 129, 36 | 421,166 |
| Scaffold 5 | Contig 67, 32, 31, 65, 25, 125, 51 | 289,808 |
| Scaffold 6 | Contig 34, 1 | 248,755 |
| Scaffold 7 | Contig 66, 69, 37 | 229,338 |
| Scaffold 8 | Contig 5, 84, 53, 90 | 209,641 |
| Scaffold 9 | Contig 2, 106, 55, 116 | 206,925 |
| Scaffold 10 | Contig 30, 78, 22 | 167,406 |
| Scaffold 11 | Contig 100, 101, 27, 114, 75, 62 | 157,464 |
| Scaffold 12 | Contig 42, 43, 89, 35 | 149,246 |
| Scaffold 13 | Contig 39, 44, 107 | 114,911 |
| Scaffold 14 | No homologous contig was detected. | 35,880 |
| Scaffold 15 | Contig 113, 165 | 28,436 |
| Scaffold 16 | Contig 104, 98, 92 | 25,582 |
| Scaffold 17 | Contig 115, 124, 127 | 23,821 |
| Scaffold 18 | Contig 118 | 13,351 |
| Scaffold 19 | Contig 123 | 8,665 |
| Scaffold 20-43 | No homologous contig was detected. |  |
