# Supplementary material for: Ecological genomics in Xanthomonas: the nature of genetic adaptation with homologous recombination and host shifts
Source: BMC Genomics. 2015 Mar 15;16(1):188. doi: 10.1186/s12864-015-1369-8 (PMC4372319; doi:10.1186/s12864-015-1369-8)
Supplement: Additional file 2: Table S2. — Information on 9 Xanthomonas species. [file 12864_2015_1369_MOESM2_ESM.doc]

**Table S2 Information on 9 *Xanthomonas* bacterial species**

1. **Basic information**

| **Species** | **Pathovar** | **Strains** | **Abbrev.** | **RefSeq ID** | **Host** | **Disease** |
| --- | --- | --- | --- | --- | --- | --- |
| ***X. citri*** |  |  |  |  |  |  |
|  | *mangiferaeindicae* | BCRC 13182 | XCM-B |  | Mango | Leaf spot |
|  | *mangiferaeindicae* | LMG 941 | XCM-L | NZ_CAHO01 | Mango | Leaf spot |
|  | *citri* | 306 | XCC | NC_003919 | Citrus | Canker |
|  | *citrumelo* | F1 | XCCM | NC_016010 | Citrus | Leaf spot |
|  | *vesicatoria* | 85-10 | XCV | NC_007508 | Pepper | Leaf spot |
| ***X. campestris*** |  |  |  |  |  |  |
|  | *campestris* | ATCC 33913 | XCCA | NC_003902 | Crucifers | Black rot |
|  | *campestris* | 8004 | XCC8 | NC_007086 | Crucifers | Black rot |
|  | *campestris* | B100 | XCCB | NC_010688 | Crucifers | Black rot |
|  | *raphani* | 756C | XCR | NC_017271 | Crucifers | Leaf spot |

1. **Genome size, number of coding sequences (CDS) and core genes**

| **Strains** | XCM-B | XCM-L | XCC | XCCM | XCV | XCCA | XCC8 | XCCB | XCR |
| --- | --- | --- | --- | --- | --- | --- | --- | --- | --- |
| **Genome size (bp)** | 5,355,324 | 5,111,537 | 5,175,554 | 4,967,469 | 5,178,466 | 5,076,187 | 5,148,708 | 5,079,002 | 4,941,214 |
| **Number of CDS** | 5362 | 4521 | 4313 | 4202 | 4487 | 4182 | 4273 | 4471 | 4535 |
| **Number of core genes** | 2851 | | | | | | | | |
