# Supplementary material for: Ecological genomics in Xanthomonas: the nature of genetic adaptation with homologous recombination and host shifts
Source: BMC Genomics. 2015 Mar 15;16(1):188. doi: 10.1186/s12864-015-1369-8 (PMC4372319; doi:10.1186/s12864-015-1369-8)
Supplement: Additional file 4: Figure S1. — Distribution of mean of K s of Xanthomonas citri pv. mangiferaeindicae BCRC 13182 (XCM-B) vs. Xanthomonas citri pv. mangiferaeindicae LMG941 (XCM-L). [file 12864_2015_1369_MOESM4_ESM.doc]

**Figure S1 Distribution of mean of *K*_s_ of *Xanthomonas citri* pv. *mangiferaeindicae* BCRC 13182 (XCM-B) vs. *Xanthomonas citri* pv. *mangiferaeindicae* LMG941 (XCM-L).**
