# Supplementary material for: Ecological genomics in Xanthomonas: the nature of genetic adaptation with homologous recombination and host shifts
Source: BMC Genomics. 2015 Mar 15;16(1):188. doi: 10.1186/s12864-015-1369-8 (PMC4372319; doi:10.1186/s12864-015-1369-8)
Supplement: Additional file 5: Figure S2. — Schematic presentation of the putative prophage region among two Xanthomonas citri pv. mangiferaeindicae (XCM) strains and Xanthomonas citri pv. citri (XCC). [file 12864_2015_1369_MOESM5_ESM.doc]

**Figure S2 Schematic presentation of the putative prophage region among two *Xanthomonas citri* pv*. mangiferaeindicae* (XCM) strains and *Xanthomonas citri* pv*. citri* (XCC).** Red arrows represent non-orthologous genes. **A.** The comparison between XCM-B and XCM-L. **B.** The comparison between XCM-B and XCC.
