# Supplementary material for: Ecological genomics in Xanthomonas: the nature of genetic adaptation with homologous recombination and host shifts
Source: BMC Genomics. 2015 Mar 15;16(1):188. doi: 10.1186/s12864-015-1369-8 (PMC4372319; doi:10.1186/s12864-015-1369-8)
Supplement: Additional file 6: Figure S3. — Comparisons of K s values between the genes with and without recombination. [file 12864_2015_1369_MOESM6_ESM.doc]

**Figure S3 Comparisons of Ks values between the genes with and without recombination.** *P* values were evaluated by Mann-Whitney U test. **A.** Two host-shifting pairs: XCC vs. *X. citri* pv. *mangiferaeindicae* BCRC 13182 (XCM-B), between citrus and mango; *X. citri* pv. *citrumelo* (XCCM) vs. *X. citri* pv. *vesicatoria* (XCV), between citrus and pepper. **B.** Two closely-related pairs: XCM-B vs. *X. citri* pv. *mangiferaeindicae* LMG 941 (XCM-L); *X. campestris* pv. *campestris* ATCC 33913 (XCCA) vs. *X. campestris* pv. *campestris* 8004 (XCC8).
